# Characterizing the Gaps Between Best-Practice Implementation Strategies and Real-world Implementation: Qualitative Study Among Family Physicians Who Engaged With Audit and Feedback Reports

**DOI:** 10.2196/38736

**Published:** 2023-01-06

**Authors:** Geneviève Rouleau, Catherine Reis, Noah Ivers, Laura Desveaux

**Affiliations:** 1 Institute for Health System Solutions and Virtual Care Women’s College Hospital Toronto, ON Canada; 2 Research Chair in Innovative Nursing Practices Centre de recherche du Centre hospitalier de l'Université de Montréal Montreal, QC Canada; 3 Institute of Health Policy, Management and Evaluation University of Toronto Toronto, ON Canada; 4 Department of Family and Community Medicine University of Toronto Toronto, ON Canada; 5 Institute for Better Health Trillium Health Partners Mississauga, ON Canada

**Keywords:** audit and feedback, family physicians, primary care, qualitative

## Abstract

**Background:**

In Ontario, Canada, a government agency known as Ontario Health is responsible for making audit and feedback reports available to all family physicians to encourage ongoing quality improvement. The confidential report provides summary data on 3 key areas of practice: safe prescription, cancer screening, and diabetes management.

**Objective:**

This report was redesigned to improve its usability in line with evidence. The objective of this study was to explore how the redesign was perceived, with an emphasis on recipients’ understanding of the report and their engagement with it.

**Methods:**

We conducted qualitative semistructured interviews with family physicians who had experience with both versions of the report recruited through purposeful and snowball sampling. We analyzed the transcripts following an emergent and iterative approach.

**Results:**

Saturation was reached after 17 family physicians participated. In total, 2 key themes emerged as factors that affected the perceived usability of the report: alignment between the report and the recipients’ expectations and capacity to engage in quality improvement. Family physicians expected the report and its quality indicators to reflect best practices and to be valid and accurate. They also expected the report to offer feedback on the clinical activities they perceived to be within their control to change. Furthermore, family physicians expected the goal of the report to be aligned with their perspective on feasible quality improvement activities. Most of these expectations were not met, limiting the perceived usability of the report. The capacity to engage with audit and feedback was hindered by several organizational and physician-level barriers, including the lack of fit with the existing workflow, competing priorities, time constraints, and insufficient skills for bridging the gaps between their data and the corresponding desired actions.

**Conclusions:**

Despite recognized improvements in the design of the report to better align with best practices, it was not perceived as highly usable. Improvements in the presentation of the data could not overcome misalignment with family physicians’ expectations or the limited capacity to engage with the report. Integrating iterative evaluations informed by user-centered design can complement evidence-based guidance for implementation strategies. Creating a space for bringing together audit and feedback designers and recipients may help improve usability and effectiveness.

## Introduction

### Background

Audit and feedback (A&F) is a quality improvement (QI) intervention that involves the collection and analysis of population- or practice-level data (audit) and the provision and delivery of clinical performance summaries (feedback) [1,2]. A&F is widely used across health care settings [3-5] by a variety of stakeholders, both to increase accountability and to improve quality of care [6]. A wide range of behaviors may be targeted, including but not limited to laboratory testing and transfusion ordering [3], adherence to clinical guidelines, and prescription [7]. Many factors influence A&F effectiveness, including the characteristics of the targeted behavior, recipients (eg, their skills and capabilities), A&F itself (eg, feedback display and delivery), and context [2,8-10]. Some targeted behaviors and contexts may be more amenable to A&F. However, all health professionals have the potential to benefit from A&F, underscoring the need to better understand whether and how to align the nature of A&F itself with the characteristics of the recipients.

Evidence indicates that the greatest effects of A&F may be achieved by optimizing the frequency of the feedback, the format in which it is delivered (verbal, written, or both), the use of visual display, the provider of the feedback (eg, a supervisor or colleague), the content of the feedback, the provision of explicit goals, and action plans [8,10-12]. Regardless of the design choices for the intervention, to change clinicians’ practice and, subsequently, patient outcomes, clinicians must first engage with A&F and then act upon the messages within.

Not all evidence-informed best practices of A&F are easily operationalized, and some may have been designed in a variety of ways (eg, color choices, positioning or size of information, or specific word choices used to describe performance). The extent to which this affects whether A&F recipients engage with it is uncertain. To address this gap, we undertook a qualitative evaluation of the redesigned *MyPractice* Primary Care report in comparison with the original report in partnership with Ontario Health (formerly Health Quality Ontario). This report includes confidential *practice profiles* that provide summary data on 3 key areas of practice: safe prescription, cancer screening, and diabetes management.

### Objectives

The initial objective of this study was to evaluate whether and how the redesign improved the usability and perceived effectiveness of the report. Early interviews challenged our underlying assumption that recipients were meaningfully engaging with the original report. We then shifted our objective to exploring the perceived usability of the report in general, with an emphasis on recipients’ understanding of the report and their engagement with it. We sought to generate recommendations on how A&F designers can work with A&F recipients to successfully operationalize best practices in the real world.

## Methods

### Ethics Approval

This project was formally reviewed by the institutional authorities of the St. Michael’s Hospital Research Ethics Board (16-076). The Women's College Hospital Research Ethics Board performed an administrative review of the study (2016-0136-E) and granted the research team an exemption from Research Ethics Board review for this study. Verbal consent to participate in the study was obtained by the interviewer (CR).

### Study Design

We conducted qualitative semistructured interviews to understand how family physicians perceived and engaged with the redesigned A&F report. We used the COREQ (Consolidated Criteria for Reporting Qualitative Research) [13] guidelines for reporting the qualitative process.

### Context and Setting

Ontario Health, an agency created by the Government of Ontario with a mandate to connect and coordinate the health care system, offers a range of resources to support health professionals in providing better care. This includes providing physicians and family health teams with information about how their practices compare with those of other physicians across the province via the *MyPractice* Primary Care report. In Ontario, primary care is delivered mainly by family physicians. The provincial health insurance plan, funded by the Government of Ontario, pays for all physician visits, tests, and prescription medications measured in the *MyPractice* Primary Care report.

### Intervention—*MyPractice* Primary Care Report

The *MyPractice* Primary Care report was initially developed by Ontario Health in partnership with the Association of Family Health Teams of Ontario, the Association of Ontario Health Centres, and the Ontario College of Family Physicians. The stakeholders involved in developing the original report were members of regulatory organizations, working primarily at the system level, knowledgeable of populational health–related data, and familiar with these types of initiatives. Physicians who were members of the cited organizations were not necessarily the end users of this report.

At the time of the study, administrative data sources were used to assess a series of quality indicators: safe prescription (eg, opioid and benzodiazepine prescription rates), cancer screening (eg, percentage of patients with up-to-date cancer screening tests for cervical, breast, and colon cancer), diabetes management (eg, percentage of patients with diabetes who had had ≥2 HbA1c tests within the past 12 months, who had diabetes and were aged >65 years and had an active statin prescription, and who had had a retinopathy screening test within the previous year), and health service use (eg, emergency department visits, hospital admissions, and readmissions [by condition]). Administrative data also encompassed clinical (chronic disease) and demographic (age and income) information on the patient population. Aggregate-level data were presented for each of the indicators, covering the previous 12 months of clinical practice. Practice improvement ideas specific to each of the topics were included to support recipients in taking action.

**Figure 1:**
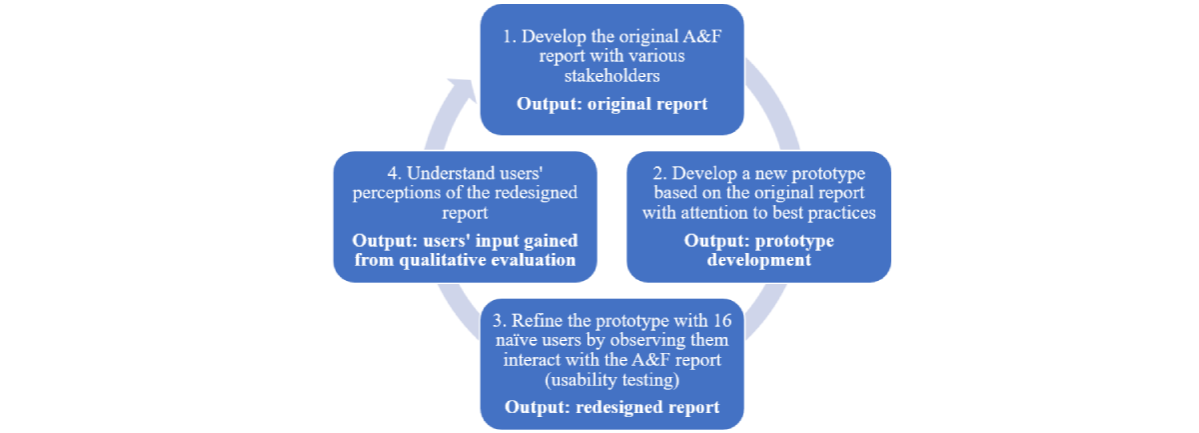
Overall development and evaluation processes of the A&F reports.

Family physicians in Ontario must sign up to receive this report. The original reports were designed without formal user testing. We developed a new prototype based on the original report, with attention to best practices [1]. We then refined the prototype with 16 naïve users (family physicians who had not signed up for the *MyPractice* report) by observing them interact with the A&F report (usability testing) with the aim of improving usability. Usability sessions involved observing participants to determine how they were navigating and understanding the A&F prototype. Participants were asked whether they were unsure about or had trouble understanding any aspects of the report and whether anything might be missing that could be helpful. The findings led to changes in the graphic design, a revised visual summary of performance compared with peers on the quality indicators, and an attempt to more clearly connect the aggregated quality indicators with suggested actions for improvement. The final product of these usability sessions was the redesigned report. Versions of the original and redesigned reports can be found in Multimedia Appendix 1. At the time of the study, to access the report, family physicians had to log in to a password-protected website. Starting in May 2017, the report was emailed to the participating family physicians. The overall development and evaluation processes of the A&F reports is presented in Figure 1.

### Participants

Eligible participants were Ontario family physicians who were registered to receive the redesigned report following its release in May 2017 and had experience with the original version. These individuals were contacted via email by Ontario Health and invited to participate in a one-time interview with a member of the research team. A CAD $100 (US $73.36) honorarium in the form of a gift card was offered. Recruitment continued until data saturation was reached. A convenience sampling approach was used whereby an email outlining the study was sent to those who registered to receive the report. Those who received the email were also encouraged to share the study information with their colleagues who had also viewed the *MyPractice* report (ie, snowball sampling).

### Data Collection

Semistructured interviews were conducted over the phone by a member of the research team (CR; see Multimedia Appendix 2 for the interview guide). The interviews were audio recorded and transcribed verbatim by an independent third party. While viewing their confidential report, participants were asked about their overall impressions of it, whether they felt it was easy to navigate, and what parts of it they found most useful and why. Participants were also asked to describe what actions (if any) they took following their review of the report (eg, conducted a chart review to determine who among their patients with diabetes was due for an HbA1c test) and what features of the report informed those actions.

### Data Analysis

The qualitative analysis followed an emergent and iterative approach. In total, 3 members of the research team (CR, NK, and BB) independently coded a first transcript; all interviews were double coded. They then met to compare the interpretation of the targeted quotes and revise the codebook. A peer debriefing was conducted—preliminary findings were discussed in a meeting with senior investigators (LD and NI) who have both conducted multiple previous qualitative studies involving A&F. The team concluded that the data were pointing to broader questions related to the perceived usability of the reports to support QI in practice. At this stage, the team reanalyzed the data using a more inductive lens. LD coded 2 transcripts to become immersed in the data. The focus of the data analysis shifted to an emphasis on recipients’ understanding of the A&F reports and their engagement with them. A conventional content analysis was performed following an inductive approach [14]. The codes and categories were iteratively revised throughout this second stage of data analysis. CR then met with another member of the research team (GR) to discuss and refine the categorization of codes and establish themes. In total, 4 members of the research team (CR, GR, LD, and NI) met to further refine the themes, which were then finalized by all authors. CR maintained a consistent audit trail of the codebook throughout the 4 stages of data analysis.

Retrospectively (ie, once the data analysis was completed), we mapped the specific redesign elements to the corresponding A&F best practice [1] they were intended to operationalize. We further mapped these applied recommendations to the corresponding theoretical constructs as outlined in Clinical Performance Feedback Intervention Theory (CP-FIT). The final themes were described with these elements to explore areas of success and failure to generate insights to optimize the real-world implementation of best-practice guidance.

## Results

### Participant Characteristics and General Interaction With A&F

A total of 17 family physicians participated in the interviews (n=8, 47% female participants and n=9, 53% male participants) lasting from 15 to 60 minutes. In total, 41% (7/17) of the participants had between 1 and 10 years of practice experience, and the remaining 59% (10/17) had >20 years in practice. Most (10/17, 59%) worked as part of a multidisciplinary family health team. Participants appreciated certain design elements such as the targeted use of color and emphasis on the number of eligible patients for a specific action as they facilitated review and interpretation of the data. This helped them better understand the data. However, family physicians described challenges in identifying actions to take in response to the data that undermined the overall utility of the report. Factors that affected the perceived usability of the report can be summarized in two key themes: (1) alignment between the report and recipients’ expectations and (2) capacity to engage with QI.

### Theme 1: Alignment Between the Report and Recipients’ Expectations Affects Usability

#### Overview of Theme 1

Family physicians described their expectations of the feedback report related to the quality indicators and data presented. First, they expected the report and its indicators to reflect best practices. Second, they expected the quality indicators to be valid and accurate. Third, family physicians expected the report to offer feedback on the clinical activities that they perceived to be within their control to change. Finally, family physicians expected the goal of the report to be aligned with their perspectives on QI. When these expectations were not met, the perceived usability of the report was low. Quotes supporting the subthemes of theme 1 are presented in Textbox 1.

**Quotes supporting the subthemes of theme 1 (alignment between the report and recipients’ expectations affects usability).**

**Subtheme 1.1: quality indicators must reflect best practices**

- “You have to make sure...numbers are important, but the number has to reflect purpose. When you give a precise number for something that’s meaningless, you have precision of something which isn’t going to motivate.” [Participant 12]
- “I’m below average for LDL testing for diabetic patients, mostly because it looks like they’re looking at me doing annual LDL testing. Personally, I think the evidence points to not actually doing this on a routine basis. And I’m at average or I’m above average with respect to statin prescriptions for those diabetic patients. So, I think that kind of fits more of what we’re trying to get at, rather than the LDL testing...I think that testing LDL doesn’t necessarily help outcomes for my patients.” [Participant 6]

**Subtheme 1.2: quality indicators must be perceived as being valid and accurate**

- “The last line that goes over the demographics is really interesting. I seem to have more of the older-age, geriatric practice and it’s kind of nice to see that because I think that influences referrals and how many times people go to the Emerg as opposed to practices that may have a much younger population. So it’s really nice that I think it acknowledges the demographic of your practice.” [Participant 11]
- “It gives a whole bunch of people that are not up to date with hemoglobin A1c testing, but it’s incorrect data. It says that most of our diabetics, I think our line is 13%, which is incorrect. So, all this stuff is not useful for me.” [Participant 8]
- “Knowing that the data is not accurate, because it’s based on [public databases]...I have less buy-in that the data actually reflects my real practice. Simply because there is no way for me to feed back to the system, either through [this report or others], to say that on this particular patient on this data point you don’t have it right.” [Participant 9]
- “Nice to have a reference of how we do compared to the rest of the province. That’s part of the thing that’s valuable, it gives us an indicator, when you get a comparator of how the provincial average is.” [Participant 15]

**Subtheme 1.3: quality indicators are expected to be actionable and within physicians’ control**

- “I think it’s in my mind more. For instance, the retinal testing I was slightly below so it was just on my mind when I’m doing my diabetic checks...It primes me to do that.” [Participant 3]
- “I get a little irritated...I mean, if you’re doing everything you can, it’s a little frustrating, because you wonder what you can do more. With these numbers, with the A1C, I see most of my diabetics every three months, so I’m thinking, well, why is that going down? Also, with the retinal scan. I mean, you have to ask them if they go to the eye doctor and they say, yes, but clearly, according to this, it’s going below the average, which means...It can be good, but it’s also frustrating, because a lot of times this is stuff out of your control...I think we have to adapt our indicator to remember that people will make their own decisions and we don’t have control.” [Participant 14]
- “I don’t think that there are many things in my control to change those numbers and so going on again and again has felt kind of like a waste of time because I’m quite sure that nothing will be different.” [Participant 16]

**Subtheme 1.4: alignment of the goal of the report with how physicians approach quality improvements**

- “I think the question I have...is what you would like physicians in general to do with the report? Because it’s all nice to give people information but if there is no clear direction about what they should do with it and how they could integrate it easily into their day-to-day use of their EMR [electronic medical record] or of their function in the office.” [Participant 9]
- “So if you’re using [data] as a guide to help physicians improve their practice that’s one thing, but if you’re using it to evaluate physicians, I think the data is just not good enough for that.” [Participant 7]
- “There’s great cancer screening, for sure, in terms of seeing where I’m at with that, seeing if, we do invest quite a bit of our staff time and energy into calling and mailing patients and reminding of that stuff. And so, to see that that’s paying off and that we’re not doing all that work and still below average or something. That’s very validating.” [Participant 5]

#### Subtheme 1.1: Quality Indicators Must Reflect Best Practices

Family physicians expected the quality indicators to reflect best practices, which for them meant alignment with the purpose of primary care, clinical guidelines, their perceptions of best practices and clinical priorities, and the realities of clinical practice. Physicians described a disconnect between the indicators and this definition of best practices and, as a result of this, a belief that the information lacked relevance to their practice, was not a priority, was not motivating, and required no action. In contrast, when there was alignment between the indicators and participants’ priorities and perceptions of clinical practice, they reported that the feedback “made sense,” was valuable, and even served to reinforce existing QI initiatives, thereby improving the perceived usability of the report.

#### Subtheme 1.2: Quality Indicators Must Be Perceived as Being Valid and Accurate

Participants wondered about the validity, accuracy, credibility, and integrity of the quality indicators and then about the data. Data are perceived as valid when the physician believes that they accurately reflect and measure the characteristics of and variations in their patient population. For a participant, the validity of the quality indicators relied on their ability to link clinical performance (eg, routine cancer screening) with huge patient outcomes (saving lives). Family physicians did not always trust the source of the data, believing them to be incorrect or outdated and leading them to trust their general perceptions over objective numbers. When the data are perceived as not useful, this negatively affects physician buy-in.

#### Subtheme 1.3: Quality Indicators Are Expected to Be Actionable and Within Physicians’ Control

When reviewing feedback in their report, physicians interpreted their current performance as reflective of either action or inaction on their part or that of their patients. Family physicians expected the report to offer feedback on the clinical activities that they perceived to be within their control to change. When family physicians determined that an indicator within the report reflected activity beyond their control, they determined that the indicator was irrelevant to their practice and did not expect to see improvement over time. Being aware of and in agreement with an area of practice requiring improvement can prime action. A major limitation of the quality indicators was the inability to capture the shared decision-making process and the person-centered approach. A physician can offer guidance and direction, but it is ultimately the patient who takes action either completing a test or receiving a treatment. Physicians expressed some frustration as the indicators were not reflective of this shared responsibility.

#### Subtheme 1.4: Alignment of the Goal of the Report With How Physicians Approach QI

Finally, family physicians were unclear as to the goal of the report and expressed a need for clearer direction or an explicit target to support action. They expected the report to be aligned with their perspective on QI: supporting point-of-care decisions by identifying areas of improvement, offering clear guidance on how to improve performance, and identifying specific targets in line with desired actions. The report was not perceived as a means of evaluating physicians’ performance as the data were not “good enough” to support this type of evaluation.

However, family physicians appreciated the opportunity to see change, specifically improvement in their performance following concrete efforts to improve.

### Theme 2: Capacity to Engage With QI Affects Usability

#### Overview of Theme 2

Even when family physicians agreed that reviewing their performance data was an important part of their professional role, they described several barriers to engaging with the report. System-level conditions (eg, time and resources) as well as work-related conditions (eg, workload and competing priorities) affected different stages of the QI process, including accessing the data, interpreting the data, and action planning. Quotes supporting the subthemes of theme 2 are presented in Textbox 2.

**Quotes supporting the subthemes of theme 2 (capacity to engage with quality improvement affects usability).**

**Subtheme 2.1: hard to fit A&F into the workflow and resource constraints**

- “We balance prevention with everything else that we do...if we followed all the good evidence in terms of prevention, and not just the things that...are in these reports...Those people, if we do what the evidence says we should do for prevention in the top ten chronic diseases, there is no time to do all the other stuff. We have to be reasonable about how we put our efforts. We could get these indicators up a lot higher, but people would be dying. It’s good that we are doing this. I’m not saying there is anything wrong with that. But the context is, this is only a tiny part of what we do. You have to look at your resources.” [Participant 13]
- “It just is one less step because if I see that I have 27 patients not tested for diabetes, I have to dangle into my EMR and do the search myself. So it’s extra searching and busy day it might not become the top of my list. But if it’s right there for me then I’m going to be more likely to follow up on that.” [Participant 3]

**Subtheme 2.2: insufficient knowledge and skills to interpret the data**

- “I’m just not sure how to interpret it. We’ll say, for example, total Emergency room visits. It tells me my practice, unadjusted, is 810 visits per 1,000 patients. Then, in the next column over, it does a risk adjustment and downgrades it to 504. I presume what that means, but I’m not entirely clear, is that my practice may be more complicated or have more comorbidities, so my number actually isn’t as bad as 810, that it’s gone down to 504 to account for that. But, again, I’m curious about that. I don’t know, does that mean I can take away from that, that I have a more complicated practice than average?” [Participant 1]
- “It’s nice to compare myself to other people but I guess what I look at, is going oh I’m doing better than everybody pretty much on everything except, you know, so now what. Just because I’m better than everybody else does that mean I’m good enough? I don’t know what that means...” [Participant 9]

**Subtheme 2.3: lack of guidance on how to prompt actions**

- “You need to be able to see how you’re doing on the big scope of things, yeah, but you need to be able to thin it down to the individual patients that make up the bigger picture. That’s what spurs the action, to identify who they are.” [Participant 2]
- “It would be nice, just to compare yourself to other people in our immediate group. I think that probably has a little more educational kind of component to it if someone is doing a lot better with something than everybody else, hey, maybe they’re doing something we can copy or emulate. It’s hard to copy people you don’t know and don’t work with and never see. So, it’s easier to engage the change idea stuff if you’ve got someone on the ground, close to you, that’s doing something different.” [Participant 2]
- “I think it’s nice to see the trend but at the same time how do we act on it now? And that’s what kind of deterred me from moving forward and using it more often. So I think our EMR would...and when we do a search we actually shoot out here are the patients who are overdue and then our nurses and team try to call those patients or keep it in the back of our minds. I think the summary is super nice to look at, out of interest, but again it’s not helping at a patient-specific level...” [Participant 11]

#### Subtheme 2.1: Hard to Fit A&F Into Workflow and Resource Constraints

Competing priorities were a reality for family physicians—they had a heavy workload of clinical tasks each day. They also had to navigate through different duties and roles as educators, leaders, and managers. Some of them reported balancing their time between preventing and treating diseases, which influenced what activities they prioritized. They had to weigh carefully how additional QI processes in response to A&F might fit into their workflow. Physicians highlighted that accessing their data, which means searching their patient records, was a time-consuming process that was hard to integrate into the existing workflow.

#### Subtheme 2.2: Insufficient Knowledge and Skills to Interpret the Data

Physicians struggled to interpret some aspects of the report and questioned the meaning of their data. Some participants clearly mentioned not knowing what to do with the aggregated practice-level numbers. A participant suggested that discussing the content of the report with someone they trusted would be helpful.

#### Subtheme 2.3: Lack of Guidance on How to Prompt Actions

Regarding action planning, physicians perceived the report as unactionable as (1) it was not perfectly up to date and (2) the aggregated nature of the data could not easily be translated into clinical actions without additional support. These challenges were not at all influenced by the visual nature of the report redesign. However, some participants appreciated the value of having their data compared with those of the rest of the province. These comparisons helped them evaluate their performance and made them aware of areas of improvement in their practice. However, the evidence-to-practice gap remained, and they did not know how to use the data to change their practice.

### Alignment Between Best-Practice Implementation Strategies and Actual Implementation

Supported by the content of Multimedia Appendix 3, we present the success and failure of our study. What seems to have worked (ie, alignment between recommendations, target of the redesign, and participants’ perspectives) was *choosing comparators that reinforce desired behavior change* and *linking the visual display and summary message*. Physicians valued the comparisons and found them helpful in pinpointing areas of improvement. However, they struggled on how to interpret the meaning of these comparators and how to consequently change their practice. Few participants commented on the visual of the feedback display, but those who did found it “nice.” This highlights a need to further understand and evolve the way *recommending actions that can improve and are under the recipient’s control* is operationalized. In the redesigned report, providing brief information regarding the importance of action on each given indicator and highlighting an absolute number of patients that appeared to require action for a given indicator did not sufficiently help recipients understand how to act. On the one hand, when physicians were in agreement with an area of practice under their control requiring improvement, it seemed to be a motivation to take action. In contrast, physicians reported that some of the indicators were beyond their control and reflected elements of care that relied on shared decision-making and patient action. Efforts in *addressing the credibility of information* and in *recommending actions consistent with established goals and priorities* were unsuccessful in promoting physicians’ engagement with the A&F report. Participants did not trust the source of the data and perceived that the information lacked relevance to their practice, was not a priority, and was not motivating. Furthermore, they did not understand how the aggregated nature of the data could translate into a way that informed clinical actions. Several physicians cited the need for cointerventions, such as peer discussion point-of-care reminders and support with action planning, to support meaningful engagement and subsequent practice change.

## Discussion

### Principal Findings

The findings suggest that, although the redesign did improve the “look and feel” of the A&F, it was not sufficient to drive practice changes in response to the data. Even if they were unconvinced that the indicators were the right targets for action, some physicians became newly self-aware of the gaps in their practice. Although this awareness may be a trigger for initiating professional behavior change processes, the desired actions for QI were unfortunately perceived as uncertain or unfeasible. The capacity to engage with the *MyPractice* report was hindered by several organizational and physician-level barriers, including the lack of fit with existing workflows, competing priorities, time constraints, and insufficient guidance and skills regarding how to interpret the data and bridge the gaps between their data and the corresponding actions.

### Lessons Learned

#### Overview

To understand the implications of the findings of this work, we applied three perspectives: (1) the recommendations by Brehaut et al [1] derived from stakeholder interviews to identify what elements of best practice need to be included in A&F interventions to improve their effectiveness; (2) the CP-FIT [2] derived from a qualitative systematic review and meta-synthesis to provide insights on how users typically progress through an A&F cycle and then help understand users based on the elements relevant to the A&F cycle (what do we need to know about users that is more important to A&F); and (3) user-centered design principles, including empathy with the end users’ goals and an understanding of their context, to operationalize those elements [12,15]. Our findings indicate the potential for integrating these perspectives into a single lens when developing and refining A&F.

#### Understanding Users, A&F Interventions, and Contextual Elements as a Whole

**Figure 2:**
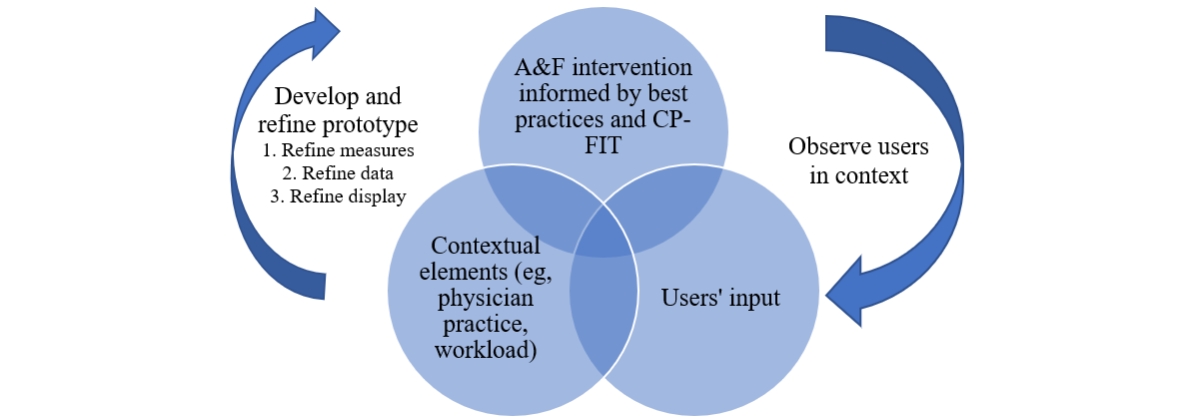
User-centered approach to develop and refine the feedback report based on a synergic understanding of users, contextual elements, and the audit and feedback (A&F) intervention (informed by Landis-Lewis et al study [12]). CP-FIT: Clinical Performance Feedback Intervention Theory.

In the refinement steps for the feedback process anchored in a user-centered design approach, Landis-Lewis et al [12] show how refining measures, data, and display can be embedded in the development and refinement step of an A&F prototype. In a learning health system approach, implementation strategy design should be iterative, informed by the ongoing collection of real-world data. Our findings highlight the importance of testing implementation strategies in context. They also echo 3 variables as proposed in the CP-FIT model [2] that influence the feedback cycle: recipient, feedback, and context variables. Figure 2 illustrates this approach to iterative A&F development following user-centered design principles that considers end users, contextual elements, and A&F intervention components.

#### Understanding the Feedback Recipients (End Users) From the Very Beginnings of Designing A&F and Seeking Out a Variety of Perspectives

It would have been appropriate to engage A&F recipients [12,15,16] from the very beginnings of designing A&F, which was not the case in this initiative. The project stakeholders (eg, Ontario Health and Association of Family Health Teams of Ontario) who designed the initial report are not the ones using the A&F report in their practice. To discover different users’ perspectives when designing the A&F intervention, it would have been useful to seek out A&F recipients with various characteristics, such as high, moderate, and low degree of exposure to A&F interventions; degree of agreement with those initiatives; and high and low performances. Although the usability sessions were conducted with naïve users, they may have focused too narrowly on the intervention elements rather than on the intervention goals. In contrast, Cooke et al [17,18] illustrated a process in which A&F report designers and physicians (end users) collaborated to design and implement A&F. The physicians identified key clinical questions, made individualized A&F reports, and developed a plan for change through participation in a group feedback session. By incorporating end-user feedback into the design of A&F, user-centered design helps ensure that the design of A&F reports is functional; can support end-user needs and goals; and, ultimately, positively influences clinical practice [12,19]. If we had used this approach, it is possible that the A&F report and recipients’ expectations would have been more aligned.

### Comparison With Prior Work

It is possible that overarching best practices for designing and implementing A&F [1,20,21] should be seen as hierarchical—some may matter more than others. For example, our study shows that, even if the “design” features of feedback display can all be addressed (eg, provide feedback in more than one way, such as presenting key messages both textually and numerically), if the focus of the A&F is not aligned with recipients’ goals and the audit itself is perceived as lacking validity, accuracy, and credibility; is poorly aligned with physicians’ priorities or not readily actionable; and is not under their control, then the intervention will not achieve its potential to improve quality. A prioritization exercise among 61 A&F stakeholders to identify the top 50 “priority” foci for the A&F research agenda [22] produced understandable variability; however, 50% of the participants identified hypotheses relating to the factors that we identified as relevant to engagement. These include testing the impact of a trusted source (“trustworthiness/credibility”), recipients being involved in the development of the feedback intervention (decision processes or conceptual model), a foundation of good-quality evidence (“trustworthiness/credibility”), and the behavior being under the control of the recipient (“self-efficacy/control”). In line with our findings, the form of the A&F reports was not extensively discussed by the participants, which led us to believe that this was not a priority for improving the effectiveness of the A&F report.

The *importance* and *relevance* of feedback goals are key variables that affect recipients’ acceptance and their intention to change their behaviors—2 key elements of the feedback cycle as outlined in the CP-FIT [2]. Family physicians in this study wanted clearer direction of what to do with the report but also clarity on the purpose and meaning of the entire A&F initiative (ie, evaluating and measuring physicians’ performance vs improving practice). Recommendations regarding the nature of desired actions further specify the need for alignment with established goals and priorities [1], which may be enhanced by including an exemplar action plan that could be adopted in response to the A&F [20]. Some family physicians thought that the quality indicators did not fairly reflect their practice and attributed the data to patient behaviors (eg, screening). In this case, physicians felt that the data represented activities beyond their control, highlighting the importance of *controllability*, which can negatively affect the acceptance of the report [2]. Physicians noted that best-practice elements of care, specifically patient-centeredness and shared decision-making, were not reflected in their data. Consequently, physicians felt judged for their performance based on data for which they were not entirely responsible, causing frustration*.* Other studies have also highlighted the need for quality indicators to reflect the important role of patient choices [6,23] as well as measures representing patients’ perspectives on care, clinical quality, and general quality of care from a broader perspective [24].

Family physicians’ views and QI knowledge and skills (or lack thereof) influence how they interact with A&F [2,23], highlighting the need for cointerventions. This corresponds to the recipient variable in the CP-FIT, specifically to *Knowledge and skills in QI*. In this study, physicians did not know how to act upon their data even though the redesigned report attempted to more closely connect the data with recommended actions (ie, “change ideas”). Educational strategies delivered alongside A&F have been effective in supporting improved adherence to guidelines [25], reducing the rate of cesarean delivery [26] and antibiotic prescription [27]. In these studies, strategies were operationalized in different ways, such as a 1-hour group session [25], quarterly educational outreach visits conducted by external facilitators [26], and 2 sessions of voluntary continuing medical education in addition to educational materials [27]. Training-based interventions effectively build skills [28] and improve communication skills [29], whereas an emphasis on data interpretation and action planning is likely to positively influence practice change [2,30]. Considering that passive feedback delivery (ie, written and delivered through email) might have played a role in physicians’ engagement, adding active interactions (eg, peer discussion or other social interactions) throughout the feedback cycle is likely necessary [18,23]. However, social interaction alone is likely to be ineffective if it does not incorporate a component of prompting actions, which may include asking targeted and reflective questions about what can change [18] or highlighting and sharing the actions of high performers [31].

These insights highlight several areas of focus as the science and implementation of A&F moves forward (Textbox 3).

**Areas of focus highlighted by insights from this study.**

**Focus areas**

- Using a user-centered design approach that considers the characteristics of and interactions between the users, their context, and the characteristics of the A&F interventions
- Engaging a variety of users (eg, current A&F users, naïve users, high performers, and low performers) to inform the development of A&F and its cointerventions
- Where resources are limited, focusing on high-value best-practice recommendations that influence engagement with the data (a necessary precursor to action and impact), including the following:
  Addressing the credibility of the data
  Including indicators that physicians value and perceive as actionable
  Recommending actions consistent with the established goals and priorities

### Limitations

First, the transferability of our findings is limited given the context, focused sample, and sampling approach (ie, 17 family physicians in Ontario who voluntarily signed up for the A&F report, including 10/17, 59% who were part of a family health team) as well as the specificity of the QI intervention examined. The way the A&F report was delivered in our study was a passive and solitary approach whereby physicians accessed their reports in confidence via email. A&F initiatives that support the creation of space for physicians to discuss the data with colleagues or a credible source and enable greater understanding and actions for improvement may be perceived as more usable. Methodologically, no member-checking process was undertaken to validate data interpretation among the research participants. However, we held peer debriefing meetings with the research team supported by senior researchers to review the data analysis and findings as well as discuss the interpretation of the findings. Finally, to address the change in research objective as mentioned previously, we described the research process in a transparent way and went back to the data to analyze and interpret them consistently to answer the research questions.

### Conclusions

This study found that esthetic design changes played a minor role in how family physicians used the A&F report. The usability of A&F appears to depend more on recipients’ perceptions of whether the quality indicators are important, accurately measured, and controllable through feasible clinical actions. Those who found the A&F report useful did so because they felt that it was aligned with the goals and priorities of their practice as a whole. Other family physicians might benefit from cointerventions to facilitate the integration of A&F into the workflow and build capacity to interpret the data and undertake practice-level actions accordingly. Health system administrators and clinicians should work together to optimize alignment between the report and the priorities of end users.

## Appendix

Multimedia Appendix 1 Screenshot of the reports.

Multimedia Appendix 2 Interview guide.

Multimedia Appendix 3 Alignment between best practices, Clinical Performance Feedback Intervention Theory (CP-FIT) constructs, target of the redesigned report, and physicians’ engagement with the report.

## Abbreviations

A&F: audit and feedback
COREQ: Consolidated Criteria for Reporting Qualitative Research
CP-FIT: Clinical Performance Feedback Intervention Theory
QI: quality improvement
