# Supplementary material for: Characterizing the Gaps Between Best-Practice Implementation Strategies and Real-world Implementation: Qualitative Study Among Family Physicians Who Engaged With Audit and Feedback Reports
Source: JMIR Hum Factors. 2023 Jan 6;10:e38736. doi: 10.2196/38736 (PMC9947922; doi:10.2196/38736)
Supplement: Multimedia Appendix 1 [file humanfactors_v10i1e38736_app1.pdf]

Multimedia Appendix 1. Original and redesigned version of *MyPractice* Report

MyPractice Report, Dashboard: Original version (Pre-redesign, May 2016)

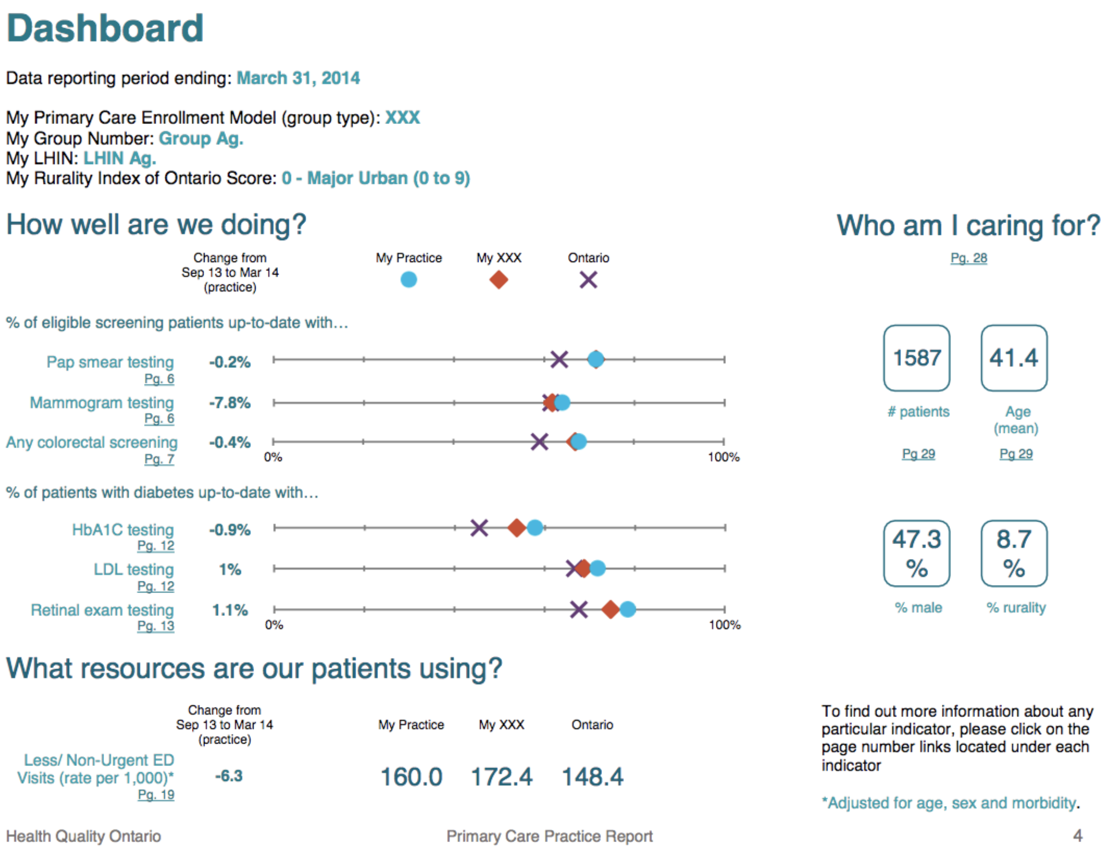

Dashboard

Data reporting period ending: **March 31, 2014**

My Primary Care Enrollment Model (group type): **XXX**
My Group Number: **Group Ag.**
My LHIN: **LHIN Ag.**
My Rurality Index of Ontario Score: **0 - Major Urban (0 to 9)**

How well are we doing?

Change from Sep 13 to Mar 14 (practice) — My Practice — My XXX — Ontario

% of eligible screening patients up-to-date with…

	Change from Sep 13 to Mar 14 (practice)
Pap smear testing Pg. 6	**-0.2%**
Mammogram testing Pg. 6	**-7.8%**
Any colorectal screening Pg. 7	**-0.4%**

0% 100%

% of patients with diabetes up-to-date with…

	Change from Sep 13 to Mar 14 (practice)
HbA1C testing Pg. 12	**-0.9%**
LDL testing Pg. 12	**1%**
Retinal exam testing Pg. 13	**1.1%**

0% 100%

What resources are our patients using?

	Change from Sep 13 to Mar 14 (practice)	My Practice	My XXX	Ontario
Less/ Non-Urgent ED Visits (rate per 1,000)* Pg. 19	-6.3	160.0	172.4	148.4

Who am I caring for?

Pg. 28

1587	41.4
# patients Pg 29	Age (mean) Pg 29
**47.3 %**	**8.7 %**
% male	% rurality

To find out more information about any particular indicator, please click on the page number links located under each indicator

*Adjusted for age, sex and morbidity.

Health Quality Ontario Primary Care Practice Report 4

MyPractice Report, Dashboard: Redesigned version (January 2017)

Primary Care Practice Report **Health Quality Ontario**

Overall Performance in Quality Indicators

Data as of March 31, 2016

	Where can I improve?	Average performance	What am I doing well?
Cancer Screening	• CRC screening	• Pap smear	• Mammogram
Diabetes Management	• HbA1C<br>• LDL	• Retinal test<br>• ACE inhibitors/ARB	• Statin
Health Services Utilization	• Total ED visits<br>• Urgent ED visits<br>• ACSC COPD	• Less urgent ED visits<br>• ACSC adm. total<br>• ACSC adm. asthma<br>• ACSC CHF<br>• ACSC diabetes	• Hospital readmissions within 30 days<br>• Hospital readmissions within 1 year<br>• Visits to own physician

View your patient information and demographics
