# Supplementary material for: Characterizing the Gaps Between Best-Practice Implementation Strategies and Real-world Implementation: Qualitative Study Among Family Physicians Who Engaged With Audit and Feedback Reports
Source: JMIR Hum Factors. 2023 Jan 6;10:e38736. doi: 10.2196/38736 (PMC9947922; doi:10.2196/38736)
Supplement: Multimedia Appendix 2 [file humanfactors_v10i1e38736_app2.pdf]

Multimedia Appendix 2. Excerpt of the interview guide questions

1. What are your overall views or impressions of the OH(Q) practice report? Please think aloud as your review it and flip through it.

   Follow-up*:* Do you feel the report is easy to navigate or intuitive?

2. What parts of the report do you find the most useful? What made them useful?
   - E.g., Dashboard overview of all indicators, individual indicator pages, data about your practice, comparison to other MDs, change ideas
3. Can you describe what action(s) you feel you can take after reviewing the report and what feature(s) inform those actions?
4. Do you currently rely on any other sources of information or feedback to monitor your performance in your practice? If so, can you describe them?
5. What changes could be made to the report to better support you in self-monitoring or achieving desired changes in your practice?

   Follow-up*:* How would these changes help support you?

6. Was this the first time you downloaded your report?

   If YES: What motivated you to access your report?

   If NO: How do you find the current layout compared to the old layout?

7. Is there anything else you would like to talk about that we haven't covered?
