# Supplementary material for: Characterizing the Gaps Between Best-Practice Implementation Strategies and Real-world Implementation: Qualitative Study Among Family Physicians Who Engaged With Audit and Feedback Reports
Source: JMIR Hum Factors. 2023 Jan 6;10:e38736. doi: 10.2196/38736 (PMC9947922; doi:10.2196/38736)
Supplement: Multimedia Appendix 3 [file humanfactors_v10i1e38736_app3.pdf]

Multimedia Appendix 3. Alignment between best practices, Clinical Performance Feedback Intervention Theory (CP-FIT) constructs, target of the redesigned report, and physicians' engagement with the report.

Best practices [a,b]	Was this a target of the redesign?	Do the findings indicate that the A&F[c] achieved those best practices and aligned with theoretical constructs?
**Delivering the feedback intervention: address the credibility of the information[c]**		
• Feedback[d]—*data collection and analysis method: 6; accuracy* [e] (acceptance[f])/ credibility and relative advantage[g]	Yes—added section to clarify the nature of data	• No<br>• Subtheme 1.1: information lacked relevance to their practice, was not a priority, and was not motivating<br>• Subtheme 1.2: participants questioned the validity, accuracy, credibility, and integrity of the quality indicators
• Feedback—*feedback delivery*: 16; function (acceptance)/compatibility	No—the only viable way to deliver to thousands of physicians was via email	• No<br>• Subtheme 1.1: information lacked relevance to their practice, was not a priority, and was not motivating<br>• Subtheme 1.2: participants questioned the validity, accuracy, credibility, and integrity of the quality indicators
• Feedback—*feedback delivery*: 17; source—knowledge and skill (acceptance)/credibility and social influence	Yes—added testimonials (quotes from other family physician recipients of the A&F)	• No<br>• Subtheme 1.2: family physicians did not always trust the source of the data
**Nature of the data available for feedback—provide feedback as soon as possible and at a frequency informed by the number of new patient cases**		

• Feedback—*feedback display*: 11; timeliness (acceptance, intention, and behavior)/actionability and credibility	No—this was a constraint of the data source and not something our team could address	• Did not emerge in the results
**Nature of the data available for feedback—provide individual rather than general data**		
• Feedback—*feedback display*: 10; specificity (acceptance, intention, and behavior) and actionability	No—only aggregate-level data were available for each of the indicators	• No<br>• Subtheme 2.3: the aggregated nature of the data could not be easily translated into clinical actions without additional support
**Nature of the data available for feedback—provide multiple instances of feedback**		
• Multiple instances of feedback are inherent to the feedback cycle	No—the frequency of feedback was constrained by the resources available at Health Quality Ontario	• Did not emerge in the results
**Feedback display—provide feedback in more than 1 way**		
• Feedback—*feedback delivery*: 18; active delivery (interaction)/compatibility and complexity	No—only written feedback	• Did not emerge in the results
**Nature of the data available for feedback—choose comparators that reinforce the desired behavior change**		
• Feedback—*feedback display*: 13; benchmarking	Yes—much more prominently highlighted which indicators were in need of improvement and revised visual summary of performance compared with peers on the quality indicators	• Yes<br>• Subthemes 1.2 and 2.3: comparisons are valuable and appreciated and helped in identifying areas of improvement. However, guidance is still needed to act upon physicians' data
• Feedback—*feedback display*: 12; trend (perception)/complexity and relative advantage	No—already present	• Yes<br>• Subthemes 1.2 and 2.3: comparisons are valuable and appreciated and helped in identifying areas of

		improvement. However, guidance is still needed to act upon physicians' data
**Nature of the desired action—recommend actions that can improve and are under the recipient's control**		
• Feedback—goal: 2; controllability (acceptance and intention)/actionability	Yes—prominently provided brief information regarding the importance of action on each given indicator	• Yes and no<br>• Subtheme 1.3: the physicians' experiences were mixed regarding *the actionability of quality indicators.* On the one hand, being aware of and in agreement with an area of practice requiring improvement can prime action (quality indicator being under physicians' control). On the other hand, some quality indicators cannot capture the shared decision-making process and the person-centered approach, and then, physicians perceive that they have no control over their indicators
• Feedback—*feedback display*: 8; performance level (intention and behavior)/actionability and compatibility	Yes—highlighted an absolute number of patients that appeared to require action for a given indicator	• Yes and no<br>• Subtheme 1.3: the physicians' experiences were mixed regarding *the actionability of quality indicators.* On the one hand, being aware of and in agreement with an area of practice requiring improvement can prime action (quality indicator being under physicians' control). On the other hand, some quality indicators cannot

		capture the shared decision-making process and the person-centered approach, and then, physicians perceive that they have no control over their indicators
**Nature of the desired action—recommend actions that are consistent with the established goals and priorities**		
• Feedback—*goal*: 1; importance (acceptance and intention)/compatibility and credibility	No—the types of quality indicators in the A&F were not in our remit	• No<br>• Subtheme 1.4: physicians are unclear about the goal of the QI[b] intervention (ie, improving vs evaluating their performance)<br>• Subtheme 2.3: the aggregated nature of the data could not easily be translated into clinical actions<br>• Subtheme 2.1: there is limited ability to interact with A&F within the existing workflows, and competing priorities (eg, preventative and curative care) limit the ability to prioritize indicators
• Feedback—*goal*: 3; *relevance* (acceptance and intention)/actionability, compatibility, and relative advantage	Yes—prominently provided brief information regarding the importance of action on each given indicator	• No<br>• Subtheme 1.4: physicians are unclear about the goal of the QI intervention (ie, improving vs evaluating their performance)<br>• Subtheme 2.3: the aggregated nature of the data could not easily be translated into clinical actions

		• Subtheme 2.1: there is limited ability to interact with A&F within existing workflows, and competing priorities (eg, preventative and curative care) limit the ability to prioritize indicators
• Context—*organization or team characteristics*: 31; workflow fit (all)/compatibility and complexity	No—understanding how users engage with A&F and their context was done later in the A&F development cycle	• No<br>• Subtheme 1.4: physicians are unclear about the goal of the QI[b] intervention (ie, improving vs evaluating their performance)<br>• Subtheme 2.3: the aggregated nature of the data could not easily be translated into clinical actions<br>• Subtheme 2.1: there is limited ability to interact with A&F within existing workflows, and competing priorities (eg, preventative and curative care) the limit ability to prioritize indicators
**Nature of the desired action—recommend specific actions**		
Context—*cointerventions*: 36; action planning (intention and behavior)/actionability, complexity, and resource match	No—deemed unfeasible at scale	• No<br>• Subtheme 2.3: all participants described challenges in understanding how to act on the data. Many highlighted the need for additional support, including point-of-care reminders (eg, through electronic medical records) and an

		educational module accompanying feedback
• Context—*cointerventions*: 35; problem-solving (perception)/actionability, compatibility, complexity, and resource match	Yes—tried to more closely and clearly connect the aggregated data with the recommended actions for improvement (ie, “change ideas”)	• No<br>• Subtheme 2.3: all participants described challenges in understanding how to act on the data. Many highlighted the need for additional support, including point-of-care reminders (eg, through electronic medical records) and an educational module accompanying feedback
• Context—co*interventions*: 34; peer discussion (perception and intention)/complexity, resource match, and social influence	No—deemed unfeasible at scale	• No<br>• Subtheme 2.3: all participants described challenges in understanding how to act on the data. Many highlighted the need for additional support, including point-of-care reminders (eg, through electronic medical records) and an educational module accompanying feedback
**Feedback display—closely link the visual display and summary message**		
Feedback—*feedback display*: 15; usability (perception)/complexity	Yes—changed the graphic design and created a declarative statement that summarized the graphical data	• Yes<br>• There are isolated qualitative data illustrating the appreciation of the A&F “look and feel”<br>• Subtheme 2.3: a participant provided positive feedback about how nice the reports are. However, they do not help prompt actions

**Feedback display—minimize extraneous cognitive load for feedback recipients**		
• Feedback—*feedback display:* 14; prioritization (perception)/complexity and relative advantage<br>• Feedback—*feedback display:* 15; usability (perception)/complexity	Yes—reducing cognitive load was a major focus	• No<br>• Subtheme 2.2: challenges in interpreting data may add extra cognitive load<br>• Subtheme 2.3: uncertainty regarding how to act on the data
**Delivering the intervention—provide short, actionable messages followed by optional detail**		
Feedback—*feedback display*: 9; patient lists (verification, acceptance, perception, intention, and behavior)/actionability, complexity, and credibility	Yes—created a declarative statement that summarized the graphical data. However, no ability to easily verify data or act upon data by reviewing patient lists	• No<br>• Subtheme 2.3: regarding action planning, physicians perceived data as unactionable, as they were out of date; they were broad and aggregated, so they did not allow physicians to inform specific courses of action, and they were not easily translated into clinical context without the appropriate support. The data prevented the physicians' capacity to engage with A&F<br>• Subtheme 1.3: however, for the few participants having appraised their data as "actionable" and as an area of improvement, they helped prompt actions. Quality indicators are expected to be actionable and within physicians' control
Feedback—*feedback display:* 14; prioritization	Yes—created a declarative statement that summarized the graphical data. However, no ability to easily verify data or	• No<br>• Subtheme 2.3: regarding action planning, physicians

(perception)/complexity and relative advantage	act upon data by reviewing patient lists	perceived data as unactionable, as they were out of date; they were broad and aggregated, so they did not allow physicians to inform specific courses of action, and they were not easily translated into clinical context without the appropriate support. The data prevented the physicians' capacity to engage with A&F<br>• Subtheme 1.3: however, for the few participants having appraised their data as "actionable" and as an area of improvement, they helped prompt actions. Quality indicators are expected to be actionable and within physicians' control
**Delivering the intervention—address barriers to feedback use**		
• CP-FIT in its entirety can be used to address barriers throughout the feedback cycle<br>• Recipient—*health professional characteristics*: 21; knowledge and skills in QI (perception, intention, and behavior)/actionability, complexity, and resource match<br>• Context—*organization or team characteristics*: 24; resources (all)/resource match	No	• No<br>• Theme 2 and its 3 subthemes: physicians described several barriers to engaging with the report. System-level conditions (eg, time and resources) as well as work-related conditions (eg, workload and competing priorities) affected different stages of the feedback cycle, including accessing the data, interpreting the data, and action planning. They did not

• Context—*organization or team characteristics*: 25; competing priorities (all)/resource match and compatibility<br>• Feedback—*feedback display*: 10; specificity (acceptance, intention, and behavior) and actionability<br>• Feedback—*data collection and analysis method*: 6; accuracy (acceptance)/credibility and relative advantage		know how to interpret the data and how to act upon their data
**Delivering the intervention—prevent defensive reactions to feedback**		
• Feedback—*feedback delivery*: *16*; function (acceptance)/compatibility	Yes—attempted to honestly summarize limitations and intended use, although it is possible that this text was missed	• Did not emerge in the results
**Delivering the intervention—construct feedback through social interaction**		
• Context—co*interventions*: 34; peer discussion (perception and intention)/complexity, resource match, and social influence	No—deemed unfeasible	• No<br>• Subtheme 2.3: participants highlighted the need for social support, including someone to help them interpret their data and identify actions and a peer champion who could role model best practices

[a] Brehaut et al’s best practices [1]
[b]CP-FIT: Clinical Performance Feedback Intervention Theory - Brown et al [2]
[c] A&F: audit and feedback
[d] CP-FIT variable category
[e] CP-FIT variable (whereas the number corresponds to the one as reported in the paper)
[f] CP-FIT feedback cycle processes
[g] CP-FIT key explanatory mechanisms
[h]QI: quality improvement.
